# Association between Serum Fatty Acids Profile and MetScore in Women with Severe Obesity

**DOI:** 10.3390/nu16203508

**Published:** 2024-10-16

**Authors:** Emilly Santos Oliveira, Fabiana Martins Kattah, Glaucia Carielo Lima, Maria Aderuza Horst, Nayra Figueiredo, Gislene Batista Lima, Renata Guimarães Moreira Whitton, Gabriel Inacio de Morais Honorato de Souza, Lila Missae Oyama, Erika Aparecida Silveira, Flávia Campos Corgosinho

**Affiliations:** 1 Postgraduate Program in Nutrition and Health, Federal University of Goiás, Rua 227, Viela Q. 68, Goiânia 74605-080, Brazil; fabiana.kattah@hotmail.com (F.M.K.); glauciacarielo@ufg.br (G.C.L.); aderuza@ufg.br (M.A.H.); gislenelima@discente.ufg.br (G.B.L.); flaviacorgosinho@ufg.br (F.C.C.); 2 Postgraduate Program in Health Sciences, Federal University of Goiás, Goiânia 74605-080, Brazil; nayra_figueiredo@discente.ufg.br (N.F.); erikasil@terra.com.br (E.A.S.); 3 Institute of Biological Sciences, Department of Physiology, USP—University of São Paulo, São Paulo 18290-000, Brazil; renata.fish@gmail.com; 4 Department of Physiology, UNIFESP—Federal University of São Paulo, São Paulo 18290-000, Brazil; gabrinacio@gmail.com (G.I.d.M.H.d.S.); lmoyama@unifesp.br (L.M.O.)

**Keywords:** severe obesity, metabolic syndrome, fatty acids, lipid profile, HOMA-IR, QUIKI

## Abstract

Background: Metabolic syndrome (MetS) is a set of conditions associated with an increased cardiovascular risk. Several serum fatty acids (FAs) seem to play an essential role in the development of cardiometabolic diseases and mortality. Thus, it is imperative to explore the impact of FAs on MetS parameters, using an early MetS screening tool such as MetScore, which is readily available in clinical practice. Aim: The aim of this study was to assess the potential correlation between serum FAs and cardiovascular risk using a MetScore. Methods: This cross-sectional study involved 41 women with severe obesity. The MetScore was calculated, and participants were categorized into high- and low-cardiovascular-risk groups based on the median MetScore value. Gas chromatography was used to quantify serum FAs. Generalized Linear Models were used to compare group means. The association was assessed through simple logistic regression, and an adjusted logistic regression was conducted to validate the association between Metscore and serum FAs. Results: The high-cardiovascular-risk group exhibited elevated values of HOMA-IR, palmitic, oleic, cis-vaccenic, and monounsaturated fatty acids, as well as the SCD-18C, indicating a heightened cardiovascular risk. Conversely, HDL-c, QUICK, gamma-linolenic, and eicosatetraenoic fatty acids showed lower values compared to the low-risk group. Conclusions: Women with severe obesity and high cardiovascular risk have lower values of some omega-3 and omega-6 FAs, considered cardioprotective and anti-inflammatory, and have higher lipogenic activity and FAs, correlated with high cardiovascular risk. These findings emphasize the need to address lipid metabolism in this population as a therapeutic target to reduce cardiovascular risk. Future research should explore clinical interventions that modulate fatty acid metabolism to mitigate cardiometabolic complications.

## 1. Introduction

Currently, obesity is one of the most frequent non-communicable diseases (NCDs) worldwide [1]. Hypertrophic adipocytes appear insulin-resistant (IR), leading to greater lipolysis and reduced lipogenesis, resulting in a heightened release of fatty acids (FAs) into the bloodstream. The high efflux of FAs in the bloodstream is one of the important triggering factors of metabolic complications such as glucose disorders, dyslipidemias, and inflammation [2]. The health implications of obesity are profound, contributing to an increased risk of several conditions such as the increased risk for cancer [3], cardiovascular disease (CVD), type 2 diabetes mellitus (DM2), and metabolic syndrome (MetS) [4].

Recent studies have shown that bariatric surgery can significantly alter the FA profile of individuals with obesity. Evaluations conducted before and after surgery indicate that, within one year, there is a notable shift towards an increase in polyunsaturated FAs (PUFAs) and a decrease in saturated FAs (SFAs) and monounsaturated FAs (MUFAs) [2,5]. These changes in FA composition are associated with a reduction in obesity-related comorbidities, including systemic arterial hypertension (SAH), DM2, and non-alcoholic fatty liver disease (NAFLD). Such findings highlight the critical relationship between FA profiles, adiposity, and metabolic disorders, suggesting that weight loss plays a pivotal role in these metabolic improvements [2,6].

MetS is a set of inter-related risk factors associated with CVD and DM2. These factors could include dyslipidemia, IR, SAH, and increased abdominal adiposity [7]. While criteria for the diagnosis of MetS already exist, the most precise strategy to establish this classification is still being discussed. The cardiovascular risk score (MetScore) was developed, aiming at a more accurate and earlier diagnosis of MetS compared to the previously recommended dichotomous classification [8].

As far as we know, no study has evaluated the relationship between serum FAs and cardiovascular risk using a MetScore, which is easily accessible for clinical practice. Since we know that the number of individuals living with severe obesity is growing worldwide [9,10] and that FA disbalance is a factor that contributes to obesity-related disorders [11], it is essential to better understand how serum FAs behave in this population and clarify their associations with cardiovascular risk. In this way, it will be possible to support public policies and guidelines that aim to improve the quality of life of these patients. Thus, the present study aims to assess the potential correlation between serum FAs and cardiovascular risk using a MetScore.

## 2. Materials and Methods

### 2.1. Participants

This analytical cross-sectional study was approved by the Research Ethics Committee of the Federal University of Goiás (protocol no 3.251.178) and by the Hospital Estadual Geral de Goiânia Dr. Alberto Rassi (protocol no 961/19).

Data collection was carried out in 2019. Patient recruitment was performed during the first consultation at the HGG outpatient clinic. Patients at this hospital outpatient clinic come from several cities in the state of Goiás and are referred by the State Health Department. During this first consultation, patients were screened according to the non-inclusion criteria. All patients who met the inclusion criteria were invited to participate in the study. Women with body mass index (BMI) above or equal to 40 (BMI ≥ 40 kg/m^2^), aged between 20 and 59 years [12] were included in the study. The non-inclusion criteria were the presence of acute inflammatory diseases, infectious diseases, neoplastic diseases, genetic syndromes, chronic consumption of alcohol (>30 g/day) and illicit drugs or psychotropic drugs, and pregnancy. All volunteers signed a written informed consent form before participating in the study.

### 2.2. Study Design

**Figure 1:**
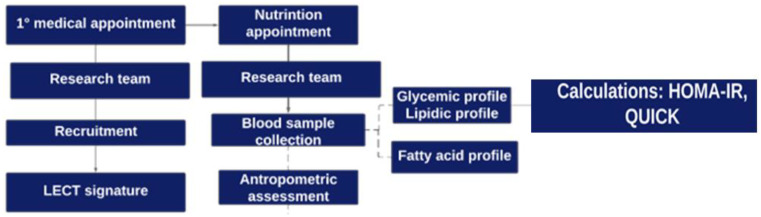
Graphical illustration of the study design: stages of participant recruitment and data collection.

Participant recruitment took place during the first consultation with the surgeon. The patients underwent anthropometric evaluation on the day of the consultation with the dietitian, and the blood sample was collected. The study design is presented in Figure 1.

### 2.3. Measurements

#### 2.3.1. Anthropometric Assessment

The anthropometric assessment was performed by a trained nutritionist, the mean values of two measurements of weight, height, neck circumference (NC), hip (HC), waist (WC), and waist–hip ratio (WHR) were calculated. The weight was measured using a Líder scale with a capacity of up to 200 kg. The volunteer was dressed in light clothes, without shoes. The circumferences were measured using an inextensible tape with a precision of 0.01 cm.

Height was measured with the patient standing and looking forward, arms extended by the sides, barefoot, leaning the back of the neck, buttocks, and heels against the wall, where the measuring tape was [13]. BMI was obtained by calculating the weight (kg) to height (m^2^) squared ratio. Abdominal circumference was measured at the largest perimeter identified between the last rib and the iliac crest at the end of expiration [14,15].

#### 2.3.2. Biochemical Exams

Qualified nurses performed blood collection through peripheral puncture. Participants fasted for at least 8 h to a maximum of 12 h before collection. By lipidogram, blood glucose and insulin were evaluated, and the glycated hemoglobin was measured by High-Performance Liquid Chromatography (HPLC). An aliquot of blood was collected and centrifuged, and the serum was stored at −80 °C until the FA dosage. The following markers of insulin resistance and sensitivity were calculated: Homeostasis Model Assessment Insulin Resistance (HOMA-IR) = Fasting insulin (µU/mL) × fasting glucose (mmol/L)/22.5 [16]; Quantitative insulin sensitivity Check Index (QUICKI) = 1/(log fasting insulin (µU/mL) + log fasting glucose (mg/dL) [17].

Based on biochemical tests, the presence of dyslipidemia was classified as follows: Isolated hypercholesterolemia (increase in total cholesterol (TC) and/or LDL-cholesterol (LDL-C)), isolated hypertriglyceridemia (increase in triglycerides (TG)), mixed hyperlipidemia (increase in of TC and TG), isolated decrease in HDL-cholesterol (HDL-C) or associated with an increase in TG or LDL-C [18].

#### 2.3.3. Blood Pressure

Blood pressure data were collected from the medical record, as measured by the nursing team before medical care.

#### 2.3.4. MetScore Calculation

The cardiovascular risk outcome variable was calculated from a proposed score [19]. The formula used the z scores of continuous metabolic risk factors (abdominal circumference, systolic blood pressure, HDL-C cholesterol, triglycerides (TG), and fasting glucose). HDL-C cholesterol and TG were log-transformed before calculating the z scores, as follows:

MetScore = (zAC + zSBP-zlogHDL + zlogTG + zfasting glucose)/5

AC = abdominal circumference; SBP = systolic blood pressure; TG = triglycerides

The patients were grouped into high- and low-cardiovascular-risk groups using the median MetScore as a cutoff point (−0.05), as there is no reference of cutoff point for this population.

### 2.4. Extraction and Determination of Serum Fatty Acids

#### 2.4.1. Extraction and Methylation of Fatty Acids

The measurement of FAs present in serum was obtained from various lipid fractions, including triglycerides, phospholipids, cholesterol esters, and free FAs. Transesterification was applied directly to the serum samples using acetyl chloride (5% HCl in methanol), which triggered the breakdown of complex lipid molecules and the release of esterified FAs. Additionally, the free FAs already present in the serum were directly methylated. All FAs resulting from this process, both free and previously esterified, were converted into FA methyl esters (FAMEs) [20,21].

#### 2.4.2. Identification and Quantification of Fatty Acid Methyl Esters (FAMEs)

The resulting FAMEs were analyzed using a Varian Model 3900 gas chromatograph (Walnut Creek, CA, USA), coupled with a flame ionization detector (FID) and an automatic sampler CP-8410. Separation was performed on a CP Wax 52 CB capillary column (Varian, Lake Forest, CA, USA) with a thickness of 0.25 μm, an internal diameter of 0.25 mm, and a length of 30 m. Hydrogen was used as the carrier gas at a linear velocity of 22 cm/s. The temperature program was set to 170 °C for 1 min, followed by increases of 2.5 °C/min until 240 °C, with a final time of 5 min. The injector and FID temperatures were maintained at 250 °C and 260 °C, respectively. The identification of FAMEs was performed by comparing the retention times of the samples with those of known standards of FA methyl esters (Supelco, 37 components; Sigma-Aldrich Darmstadt, Alemanha; Mix Me93, Larodan; and Qualmix, PUFA Fish M, Menhaden Oil, Larodan). The concentrations of fatty acids were expressed as percentages of the total fatty acids present [21].

### 2.5. Desaturases Indexes

The desaturase indexes can demonstrate the indirect action of stearoyl-CoA desaturase 1 (SCD1) activity, indicating de novo *lipogenesis* of MUFAs (C16:1 and C18:1) from the SFAs (C16:0 and C18:0). The index was obtained by the ratio of fatty acids 16:1n[7]/16:0 (palmitic FA desaturase index (SCD-16)) and 18:1n[9]/18:0 (oleic FA desaturase index (SCD-18)) [22].

### 2.6. Statistical Analysis

Statistical analyses were performed using the Statistical Software Package for the Social Sciences (SPSS) (version 25; SPSS Inc., Chicago, IL, USA). Initially, a total of 45 volunteers were recruited for the study; however, we experienced a sample loss of 4 participants (approximately 9%), primarily due to incomplete data collection (2 participants) and failure to comply with fasting requirements for the exams (2 participants). In the end, the study included 41 participants.

The sample size calculation was carried out, based on Pearson’s correlation test, between the variables, assuming an α (Rho) of 0.50 and with Power (1 − β) = 0.80 of 0.05; the sample size necessary to validate these analyses is 41 individuals. The Shapiro–Wilk test was used to verify data distribution. The variables studied are described as mean and standard deviation (SD). Individuals were grouped based on the median of the outcome variable (MetScore) into high- and low-cardiovascular-risk groups, and the cutoff value was −0.05. The difference between group means was assessed using the Generalized Linear Model (GLzM). Spearman and Spearman’s coefficients were utilized to verify the MetScore correlations with the other variables. The chi-square test evaluated the relationship between medication intake and cardiovascular risk. Simple logistic regression was used to verify the association between the outcome and the other variables. In addition, a logistic regression model adjusted for age, height, HOMA-IR, HDL-c, and use of hypoglycemic agents was also developed to verify the association between cardiovascular risk and FAs. The association measure for the regressions was the odds ratio with its respective 95% Confidence Interval. The significance level was set at <0.05.

## 3. Results

### 3.1. Sample Description and Lipidic, Glycemic Profile

A total of 41 individuals with a mean age of 40.2 ± 8.31 years and a mean BMI of 48.38 ± 6.66 kg/m^2^ were included in the study following the inclusion criteria. All individuals were classified with increased risk for the development of cardiovascular diseases according to AC, WHR, and NC. About the use of medications, there was no statistical difference between the low- and high-cardiovascular-risk groups. The reference values for all mentioned variables can be found in the footnote of Table 1.

Regarding the lipid profile, 78.05% of the volunteers had some dyslipidemia, in which 39.02% had isolated hypercholesterolemia, 31.70% had mixed hyperlipidemia, and 7.31% had just hypertriglyceridemia; 60.97% had low HDL-c, and 21.95% had no dyslipidemia. Twenty women (48.78%) presented associated dyslipidemia, consisting of low HDL-c with another type of dyslipidemia. Despite this, only 1 patient reported using statins.

The group with the highest risk was older (*p* = 0.042), had higher HOMA-IR (*p* = 0.011), HbA1c (*p* = 0.002) and VLDL-C (*p* ≤ 0.001) and QUICKI (*p* = 0.006). The clinical data of the patients can be observed in Table 1.

### 3.2. Serum Fatty Acid Profile and Their Association with Cardiovascular Risk

Considering the serum fatty acid profile, 23 FAs were identified, and all were included in the study. Among the 23 FAs identified, six were SFAs (56.18%), twelve were PUFAs (26.64%), and five were MUFAs (17.15%). When evaluating the abundance of omega-3 and 6 fatty acids, these presented averages of 4.96% and 21.68%, respectively. The mean anti-inflammatory omega 3/6 ratio was 0.24, whereas the pro-inflammatory omega 6/3 ratio was 5.23. Table 2 shows the percentage of each FA evaluated in participants.

Considering the FA profile, the high-risk group had the greatest % of oleic (C18:1n[9]) (*p* = 0.005), cis-vaccenic (c18:1n[7]) (*p* = 0.042), and total MUFAs (*p* = 0.002). There was also a lower % of gamma-linolenic (C18:3n[6]) (*p* = 0.003) and eicosatetraenoic acid (C20:4n[3]) (*p* = 0.038). All the differences found between groups were adjusted by age. As for the desaturase indexes, the group with high cardiovascular risk showed greater activity of SCD-18 (*p* = 0.011). There was no statistically significant difference in the SCD-16C; these data are presented in Table 2.

Logistic regression analysis showed a significant association between cardiovascular risk calculated by the MestScore and glycemic and lipid parameters in women with severe obesity. Furthermore, every increase of one unit of palmitic FA increases the chance of belonging to the high-cardiovascular-risk group by 20%. As for total MUFAs, the chance of belonging to the high-cardiovascular-risk group increases by 42% with an increase of one unit of the total MUFAs and by 27% with an increase in FA c18:1n[9]. The analysis of PUFAs showed that every decrease of one FA C18:3n[6] unit increases the chance of belonging to the high-cardiovascular-risk group by 63%. We also observed that an increase of one unit in SCD-18 increases cardiovascular risk by 673%; these data are presented in Table 2.

The logistic regression model adjusted for each statistically significant FA (*p* ≤ 0.05) included age, height, HOMA-IR, HDL-c, and use of hypoglycemic agents as adjustment covariates. The adjusted OR comparing cardiovascular risk estimates for women with severe obesity remained statistically significant for all variables except SCD-18C, Table 3.

The analysis of c16:0 (*p* = 0.030 OR = 1.36), total MUFAs (*p* = 0.032 OR= 1.52), and C18:1n[9] (*p* = 0.045 OR = 1.36) showed that, with each increase of 1 unit of these serum FAs, there is an increase in the individual’s belonging to the high-cardiovascular-risk group of 36%, 52%, and 36%, respectively. As for FA C18:3n[6], it can be detected that every increase of 1 unit reduces the chance of belonging to the high-cardiovascular-risk group by 73%.

Correlations were observed between the MetScore and metabolic parameters, presented in Table 4.

## 4. Discussion

Identifying the associations between plasma FAs and cardiovascular risk can support the development of scientific knowledge about the influence of FAs on the cardiometabolic health of individuals with obesity. This association is crucial since plasma FAs play an important role in the metabolic pathways that influence insulin resistance, lipid metabolism, and inflammation. Together, these changes are fundamental in the development of cardiometabolic dysfunction associated with obesity. Addressing these factors may provide information on therapeutic targets to reduce cardiovascular risk in this vulnerable population. In the present study, for the first time, we have identified that women with severe obesity and high cardiovascular risk presented higher proportions of the SFA C16:0, a greater amount of SCD-18 and total MUFAs, and lower proportions of C20:4n3 and C18:3n6, suggesting the worst lipid profile.

In addition, the data also indicate that every increase of one unit of FA C16 increases the individual’s chance of belonging to the high-risk group by 20%. Long-chain fatty acids may contribute to the progression of obesity once they are less oxidized than other types of FAs [11]. Previous studies have demonstrated the relationship between palmitic FAs and cardiovascular health in different populations [11,23,24]. Among the mechanisms by which palmitic acid correlates with worse cardiometabolic outcomes, we highlight its effect on promoting oxidative stress, increasing reactive oxygen species, and the activation of inflammatory signaling [25,26], which favors impairment in hepatic glucose and lipid metabolism, fomenting IR, atherosclerosis, and hypertriglyceridemia [27,28].

In the present study, MUFAs corresponded to an average of 17.15% serum FAs with a higher percentage of total MUFAs in individuals with high cardiovascular risk compared to those with low risk. In an analysis adjusted for age, height, HOMA_IR, and HDL-c, MUFAs remained an independent predictive factor for cardiovascular risk, being able to increase the risk by 39% (Table 3). Wrzosek et al. found similar results in a sample of both women and men with obesity, where higher amounts of serum oleic FAs were found in the group with lower amounts of total PUFAs (considered a worse profile for cardiovascular risk) [2]. These data may be seen as controversial since the dietary intake of MUFAs is associated with reduced cardiometabolic risk. However, it is known that plasma MUFAs do not represent only dietary intake, as they are affected by genetic factors, smoking, physical activity, and especially by de novo *lipogenesis* that transforms surplus SFAs into MUFAs through the enzymes SCD-1 [29,30].

SCD-1 is a desaturase found mainly in the liver and adipose tissue, responsible for converting a portion of 16:0 into palmitoleic (16:1) and 18:0 into oleic acid (18:1), which might contribute to the higher abundance of MUFAs. Considering that SCD-1 is regulated by factors such as saturated fat intake, carbohydrate consumption [31], and metabolic conditions, its elevated activity is associated with metabolic diseases such as insulin resistance, obesity, type 2 diabetes, and cardiovascular dysfunction, since the excessive production of MUFAs can lead to the accumulation of fat in the liver and adipose tissue, favoring the development of cardiometabolic complications, which have been associated with obesity, IR, DM2, and MetS [32,33].

A recent study found that SCD-1-deficient mice are protected against obesity, MetS, CVD, and NAFLD [34,35,36]. In addition, those animals showed greater thermogenesis and insulin sensitivity [37], although the mechanisms are not completely elucidated. In the studied population, the ability of SCD-18 to increase cardiovascular risk was estimated at 673% for each unit increased in the index (*p* = 0.023). In agreement, Svendsen et al. obtained significant values of SCD-18, but not of SCD-16, in individuals categorized to be at high cardiometabolic risk [38]. These findings can be attributed to excessive dietary intake of SFAs, simple carbohydrates, and other unhealthy eating habits [32,38] highlighting the importance of guidelines and nutritional guides that address the consumption of fatty acids, simple carbohydrates, and their consequences for health in a practical and easy-to-understand way for the general population.

The activity of desaturases explains the higher proportion of oleic FA (*p* = 0.005) and vaccenic FA (*p* = 0.042) in women with high cardiovascular risk. Each unit of oleic FA was associated with a 27% increase in cardiovascular risk (Table 2). Both FAs were also negatively correlated with insulin sensitivity (*p* = 0.002, r = −0.477; *p* = 0.013, r = −0.389, respectively), showing their effect on glucose metabolism. Previous studies point out oleic and vaccenic fatty acids as pro-obesogenic and diabetogenic due to their ability to promote IR [38]. Therefore, the hypothesis of endogenous synthesis of MUFAs as one of the etiological factors of glycemic disorders is strengthened [39,40]. However, further studies are needed to investigate the pathophysiological mechanisms [41,42].

De novo lipogenesis-produced fatty acids, including palmitic acid (16:0), cis-palmitoleic acid (16:1*n*-7), oleic acid (18:1*n*-9), and cis-vaccenic acid (18:1*n*-7), are associated with risk factors for cardiovascular diseases, such as adiposity, hypertension, diabetes, inflammation, and cardiovascular mortality [27,40,41,43]. The cis-vaccenic fatty acid can reduce insulin sensitivity through various mechanisms. This fatty acid can activate serine/threonine kinases, which, in turn, decrease the phosphorylation of tyrosine residues on insulin receptor substrates (IRS1/2). This impairs the IRS/phosphatidylinositol 3-kinase (PI3K) signaling pathway, hindering insulin signal transduction and contributing to insulin resistance. Furthermore, free fatty acids, such as cis-vaccenic acid, may lead to the generation of reactive oxygen species and act as modulators of the NLRP3 inflammasome, which may play an important role in insulin resistance [27,31,43].

A recent study showed that women with polycystic ovary syndrome (PCOS) had higher concentrations of cis-vaccenic acid [44]. However, further interventional studies are needed to investigate whether reducing the lipogenesis of vaccenic acid, through dietary or pharmacological interventions, could positively impact insulin sensitivity and cardiovascular outcomes, as this pathway is not entirely clear. Understanding these pathophysiological mechanisms could pave the way for new therapeutic strategies [44].

It is known that IR is closely related to cardiometabolic risk [45], being a parameter that increases the MetScore. The mean values of fasting blood glucose, HbA1c, HOMA-IR, and QUICK, indicate that the patients have disorders in glucose metabolism [16,17,46]. The group with high risk presented worse glucose parameters. In addition, we found that MetScore correlated with HOMA-IR (*p* = 0.001, r = 0.481), HbA1c (*p* = 0.00, r = 0.526), and QUICK (*p* = 0.005, r = −0.430). Furthermore, MetScore also correlated negatively with HDL-C (*p* = 0.001, r = −0.514) and omega 6 (*p* = 0.004, r = −0.313), suggesting an intrinsic pathway between glucose lipid metabolism. Finally, the high-risk group presented lower HDL-c (*p* = 0.008) and higher VLDL-c (*p* < 0.001).

Regarding the PUFA profile, data revealed an average of 26.64%, of which 21.68% belonged to the omega-6 family and 4.96% from the omega-3 family. Wrzosek et al. found a similar fatty acid profile, with 31.23% of the total PUFAs being 25.92% omega-6 and 1.78% omega-3 [2]. The total PUFAs showed no statistical difference between the groups. However, FA C18:3n[6] (gamma-linolenic) had lower proportions in the high-risk group (*p* = 0.003). It was also observed that a reduction of 1 unit of GLA increases the chance of belonging to the high-risk group by 63% (Table 2). Moreover, we observed a negative correlation between MetScore and GLA (Table 4). Moderate intake of linoleic acid, in partial replacement of SFAs, was also associated with reduced cardiometabolic risk due to reduced LDL-c through upregulation of the hepatic LDL-c receptor gene, thus leading to clearance of circulating LDL-c [47].

**Figure 2:**
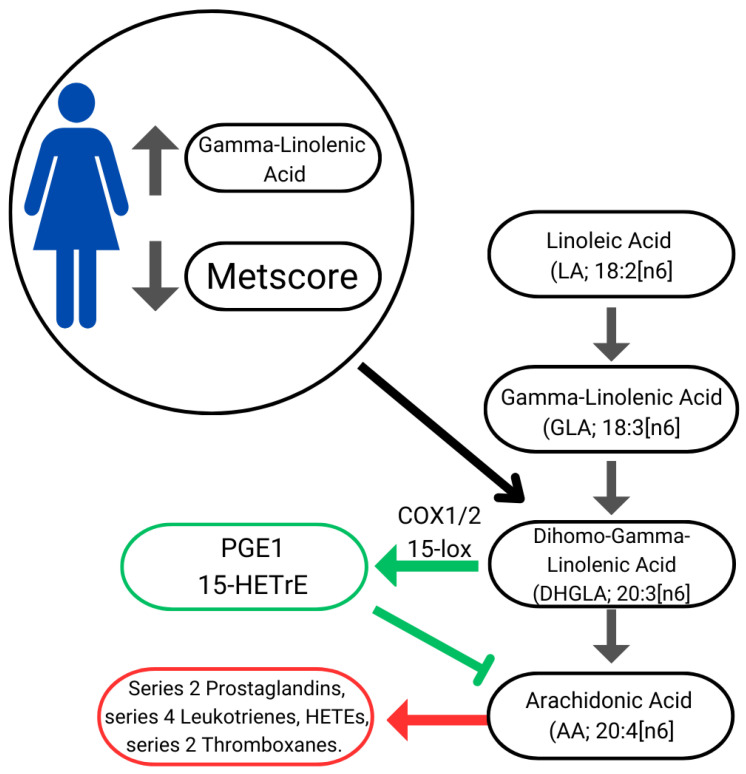
Illustration of linoleic acid bioconversion pathway, eicosanoid synthesis, and cardiovascular risk. Green arrows: anti-inflammatory pathways; Red arrow: inflammatory pathways.

Lower cardiovascular risk may also be associated with higher GLA values due to its beneficial health effects arising mainly from the conversion of GLA into Dihomo-Gamma-Linolenic (DHGLA) which gives rise to metabolites such as cyclooxygenase 1/2 (COX1/2). The COX1/2 increases prostaglandin series 1 (PGE1) and 15-lipoxygenase (15-Lox) forming 15-(S)-hydroxy-8,11,13-eicosatrienoic acid (15-HETrE). DHGLA-derived metabolites suppress inflammation, promote vasodilation, lower blood pressure, and exert antineoplastic effects [38]. Furthermore, both PGE1 and 15-HETrE antagonize the synthesis of pro-inflammatory metabolites derived from Arachidonic Acid (AA), such as series 2 Prostaglandins, series 4 Leukotrienes, HETEs, and series 2 thromboxanes (Figure 2) [48]. GLA is commonly available as a dietary supplement; however, its efficacy in attenuating inflammation through dietary intake remains controversial [49,50,51]. Further research is required to clarify its therapeutic potential in this context [52].

Women that were grouped in the high-risk group also presented lower proportions of eicosatetraenoic acid (C20:4n[3]) (*p* = 0.038), corroborating previous findings [53,54]. A recent meta-analysis with a cross-sectional and case–control study identified that higher levels of omega-3 PUFA were associated with a lower prevalence of MetS [53]. Another meta-analysis with prospective cohort studies found higher dietary and plasma omega 3 PUFA levels associated with a significantly low risk of developing MetS [54].

**Figure 3:**
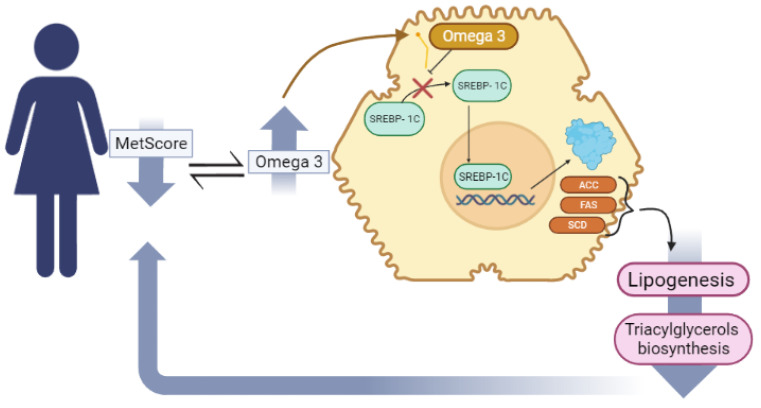
The mechanism by which PUFA [n3] decreases triacylglycerol biosynthesis.

The possible mechanisms for the protective effects of omega 3 PUFA on MetS are related to reduced synthesis of triacylglycerols and LDL-c in the liver, preventing dyslipidemias [55]. Omega-3 can reduce the synthesis of fatty acids in the liver by suppressing the nuclear abundance of the transcription factor SREBP-1c that promotes the activation of essential genes in the regulation of lipogenic enzymes, acetyl-CoA carboxylase (ACC) and fatty acid synthase (FAS), which are responsible for catalyzing lipogenesis, increasing the efflux of serum FFAs for the formation of triacylglycerols (Figure 3) [55,56].

The omega-3 PUFAs also play a role in improving insulin sensitivity^36^ and in controlling arterial hypertension as they can reduce the levels of the angiotensin-converting enzyme [57]. However, in the present study, we did not find a correlation between omega 3 PUFAs and glycemic parameters.

Although serum fatty acids are not analyzed in clinical practice, it is essential to understand how they favor cardiovascular health or disease. For the first time in the literature, through the present study, it was possible to demonstrate in women with severe obesity the association of critical fatty acids favoring cardiovascular risk, suggesting that greater attention should be given to nutritional guidelines.

Among the limitations of the study, we mention the fact that the patient’s food context was not analyzed. However, other studies that addressed the same topic did not observe significant differences between dietary intake and the profile of serum fatty acids in relation to cardiovascular risk [2]. Furthermore, it is necessary to carry out longitudinal studies with a larger sample to strengthen the findings of this study.

As strengths, we highlight the use of MetScore for early screening of cardiovascular risk and its use in association with FAs. In addition, the scientific literature lacks data on patients with severe obesity, which requires special attention and a better understanding of the cardiovascular risk to which they may be exposed.

## 5. Conclusions

Women with severe obesity who have a high cardiovascular risk calculated by MetScore tend to have a worse FA profile, with lower amounts of GLA and FA C20:4n[3], which are considered cardioprotective and anti-inflammatory, and higher percentages of palmitic FA and serum MUFA, which are associated with inflammation and CVD. They also have higher de novo lipogenic activity due to the conversion of SFAs to MUFAs, which is associated with the increased cardiovascular risk demonstrated by the indices of desaturases. These findings emphasize the need to address lipid metabolism in this population as a therapeutic target to reduce cardiovascular risk. Future research should explore clinical interventions that modulate fatty acid metabolism to mitigate cardiometabolic complications.

## 6. Future Recommendations

We recommend that future research focus on the following points:

(1) There is a need for longitudinal studies to determine the influence of fatty acids on cardiovascular risk over the years.
(2) Clinical trials for dietary interventions should be conducted using different sources of carbohydrates and lipids to verify the influence of serum fatty acids and desaturating enzymes on cardiovascular risk. Furthermore, randomized clinical trials with fatty acid supplementation, which in the present study was shown to be cardioprotective, would help determine their influence on reducing cardiovascular risk.
(3) Evaluation of different populations, mainly longitudinal studies in eutrophic individuals, should be conducted to show the MetS score as a good tool for early screening of cardiovascular risk, through changes in the GA profile over the years.
(4) It is important to conduct controlled and randomized clinical trials involving the supplementation of key fatty acids discussed in this study in order to investigate their potential as a therapeutic option for attenuating inflammation and reducing cardiometabolic alterations.

## Figures and Tables

**Table 1:** Association between high cardiovascular risk and clinical, anthropometric, and laboratory variables in women with severe obesity.

Variable	Mean Total Sample ± SD	Low Risk (<−0.05) Mean ± SD	High Risk (≥−0.05) Mean ± SD	*p* ^1^	High Risk OR (95% CI)	*p* ^2^
Age (years)	40.22 ± 8.31	37.65 ± 9.10	42.67 ± 6.83	0.042 *	1.08 (0.99–1.18)	0.058
Height (m)	1.59 ± 0.05	1.60 ± 0.05	1.58 ± 0.05	0.167	5.21^−4^ (7.25^−29^–37.4)	0.185
Weight (kg)	122.71 ± 17.98	124.33 ± 31.30	121.17 ± 16.76	0.551	0.99 (0.96–1.03)	0.571
BMI (kg/m^2^)	48.38 ± 6.66	48.22 ± 6.89	48.53 ± 6.60	0.878	1.05 (0.91–1.11)	0.881
AC (cm)	131.46 ± 12.52	127.33 ± 9.54	135.41 ± 13.91	-	-	-
HC (cm)	145.32 ± 13.33	145.41 ± 14.30	145.25 ± 12.70	0.967	0.99 (0.95–1.05)	0.968
WHR	0.90 ± 0.06	0.88 ± 0.07	0.93 ± 0.05	-	-	-
NC (cm)	41.63 ± 3.23	41.50 ± 3.77	41.77 ± 2.71	0.789	1.03 (0.85–1.24)	0.791
Fasting glucose (mg/dL)	110.87 ± 35.13	95.25 ± 8.60	125.76 ± 43.83	-	-	-
HbA1c (%)	6.24 ± 1.10	5.82 ± 0.36	6.65 ± 1.39	0.002 *	5.24 (1.01–27.05)	0.048 *
HOMA-IR	6.92 ± 3.48	5.64 ± 2.60	8.15 ± 3.82	0.011 *	1.30 (1.02–1.64)	0.031 *
QUICKI	0.28 ± 0.24	0.30 ± 0.02	0.28 ± 0.02	0.006 *	3.86^−19^ (6.17^−35^–0.00)	0.022 *
Insulin	27.55 ± 14.42	27.7 ± 16.14	27.40 ± 12.99	0.945	0.99 (0.96–1.04)	0.943
TG (mg/dL)	145.17 ± 68.19	108.70 ± 36.50	179.91 ± 73.75	-	-	-
LDL-C (mg/dL)	105.4 ± 28.21	108.80 ± 32.44	101.86 ± 23.82	0.418	0.99 (0.98–1.01)	0.429
HDL-C (mg/dL)	46.75 ± 10.31	50.65± 10.84	43.05 ± 8.46	-	-	-
VLDL-C	21.00 ± 5.06	21.00 ± 5.06	29.24 ± 9.76	0.000 *	1.23 (1.06–1.41)	0.005 *
SBP (mmHg)	138.68 ± 15.09	132.25 ± 14.29	144.81 ± 13.46	-	-	-
DBP (mmHg)	86.02 ± 9.93	82.80 ± 9.83	89.10 ± 9.23	0.032	1.08 (0.99–1.18)	0.059
MetScore	−0.10 ± 0.51	-	-		-	-
Medications in use (n (%))						
Antihypertensive				0.437 ^3^	-	-
Yes	21 (51.2%)	9 (22.0%)	12 (29.3%)			
No	20 (48.8%)	11 (26.8%)	9 (22.0%)			
Hypoglycemic agents				0.089 ^3^	-	-
Yes	6 (14.6%)	1 (2.4%)	5 (12.2%)			
No	35 (85.4%)	19 (46.3%)	16 (39.0%)			
Statins				0.300 ^3^	-	-
Yes	1 (2.4%)	1 (2.4%)	0 (0%)			
No	40 (97.6%)	19 (46.3%)	21 (51.2%)			

BMI: body mass index. AC: abdominal circumference (<80 cm); NC: neck circumference (<34 cm); HC: hip circumference; WHR: waist–hip ratio; Glycemia (between 70 and 99 mg/dL); HbA1c: glycated hemoglobin (<5.7%); HOMA-IR: Homeostasis Model Assessment Insulin Resistance (<2.71); QUICKI: Quantitative insulin sensitivity Check Index (≥0.321); Insulin (between 2.6 and 24.9); TG: Triglycerides (<150 mg/dL); LDL-C: low-density lipoprotein (<130 mg/dL); HDL: high-density lipoprotein (>40 mg/dL); SBP: systolic blood pressure (120 mmHg); DBP: Diastolic Blood Pressure (90 mmHg); *p* ^1^: *p*-value of difference between means using GzLM; *p* ^2^: *p*-value of logistic regression. OR: odds ratio; ^3^: chi-square test. *: statistically significant difference.

**Table 2:** Analysis of difference between means and logistic regression of fatty acids in women with severe obesity.

Variable (MetScore)	Mean (%) ± SD	Low Risk (<−0.05) Mean ± SD	High Risk (≥−0.05) Mean ± SD	*p* ^1^	High Risk OR (95% CI)	*p* ^2^
SFA total %	56.18 ± 5.63	56.89 ± 4.78	55.51 ± 6.39	0.417	0.96 (0.85–1.07)	0.432
C12:0 (lauric)	26.42 ± 8.35	28.61 ± 7.18	24.35 ± 9.02	0.089	0.94 (0.86–1.01)	0.109
C14:0 (myristic)	4.26 ± 1.45	4.69 ± 1.27	3.87 ± 1.54	0.058	0.65 (0.41–1.05)	0.076
C16:0 (palmitic)	15.61 ± 4.75	13.77 ± 3.75	17.38 ± 5.03	0.008 *	1.20 (1.03–1.40)	0.020 *
C18:0 (stearic)	9.18 ± 1.05	9.08 ± 1.04	9.28 ± 1.08	0.526	1.21 (0.67–2.20)	0.530
C20:0 (arachidic)	0.55 ± 0.14	0.59 ± 0.12	0.52 ± 0.16	0.108	0.03 (3.12^−4^–2.64)	0.124
C22:0 (behenic)	0.13 ± 0.13	0.16 ± 0.13	0.12 ± 0.13	0.369	0.118 (0.01–13.25)	0.375
MUFA%	17.15 ± 2.99	15.84 ± 1.87	18.41 ± 3.35	0.002 *	1.42 (1.09–1.86)	0.011 *
C14:1n[5] (myristoleic)	3.17 ± 1.01	3.47 ± 0.90	2.90 ± 1.06	0.058	0.55 (0.28–1.07)	0.078
C16:1n[7] (palmitoleic)	0.90 ± 0.24	0.95 ± 0.23	0.86 ± 0.26	0.225	0.22 (0.02–2.99)	0.253
C18:1n[9] (oleic)	11.23 ± 3.92	9.65 ± 2.62	12.75 ± 4.40	0.005 *	1.27 (1.04–1.43)	0.017 *
C18:1n[7] (vacenic)	1.11 ± 0.35	1.00 ± 0.31	1.22 ± 0.37	0.042 *	6.43 (0.90–45.52)	0.062
C20:1n[9] (eicosenoic)	0.72 ± 0.22	0.77 ± 0.21	0.68 ± 0.24	0.238	0.19 (0.01–3.11)	0.243
Total PUFA (%)	26.64 ± 4.04	27.25 ± 3.76	26.07 ± 4.30	0.338	0.93 (0.79–1.09)	0.350
Omega 6 total %	21.68 ± 4.41	22.06 ± 3.86	21.33 ± 4.95	0.589	0.96 (0.84–1.11)	0.592
C18:2n[6] (LA; linoleic)	14.47 ± 5.16	14.38 ± 4.73	14.57 ± 5.66	0.906	1.01 (0.89–1.14)	0.906
C18:3n[6] (GLA; gamma-linolenic)	3.45 ± 1.05	3.90 ± 1.08	3.03 ± 0.86	0.003 *	0.37 (0.16–0.82)	0.015 *
C20:2n[6] (eicosadienoic)	1.73 ± 0.69	1.85 ± 0.76	1.63 ± 0.62	0.275	0.62 (0.24–1.59)	0.317
C20:4n[6] (AA; arachidonic)	0.44 ± 0.78	0.61 ± 1.33	0.28 ± 1.07	0.172	0.52 (0.17–1.53)	0.234
C20:3n[6] (DGLA; dihomo-gamma-linolenic)	0.32 ± 0.99	0.07 ± 0.30	0.57 ± 1.33	0.089	2.21 (0.66–7.36)	0.198
C22:2n[6] (docosadienoic)	1.25 ± 0.55	1.26 ± 0.56	1.25 ± 0.57	0.924	0.95 (0.31–2.89)	0.924
Omega 3 total%	4.96 ± 1.99	5.19 ± 1.71	4.75 ± 2.25	0.455	0.89 (0.65–1.22)	0.469
C18:3n[3] (ALA; alfa-linolenic)	2.78 ± 1.30	3.03 ± 1.18	2.54 ± 1.41	0.180	0.73 (0.44–1.22)	0.234
C18:4n[3] (stearidonic)	0.50 ± 0.24	0.49 ± 0.12	0.52 ± 0.33	0.649	1.64 (0.12–22.04)	0.708
C20:3n[3] (eicosatrienoic)	0.76 ± 0.69	0.69 ± 0.67	0.83 ± 0.72	0.413	1.36 (0.54–3.44)	0.517
C20:4n[3] (eicosatetraenoic)	0.27 ± 0.11	0.31 ± 0.07	0.24 ± 0.14	0.038 *	0.002 (2.09^−6^–1.53)	0.066
C20:5n[3] (EPA; eicosapentaenoic)	0.08 ± 0.32	0.03 ± 0.11	0.13 ± 0.45	0.348	3.63 (0.13–97.89)	0.443
C22:6n[3] (DHA; docosahexaenoic)	0.55 ± 0.56	0.63 ± 0.68	0.48 ± 0.44	0.398	0.60 (0.17–2.11)	0.426
omega 3/6 ratio	0.24 ± 0.13	0.25 ± 0.10	0.25 ±0.17	0.930	1.21 (0.02–107.11)	0.934
omega 6/3 ratio	5.23 ± 2.51	4.82 ± 2.07	5.63 ± 2.87	0.307	1.14 (0.89–1.47)	0.301
SFA/PUFA Ratio	2.19 ± 0.63	2.15 ± 0.50	2.24 ± 0.75	0.606	1.26 (0.47–3.42)	0.645
SFA/MUFA ratio	3.41 ± 0.89	3.66 ± 0.69	3.18 ± 1.01	0.061	0.512 (0.24–1.11)	0.091
SCD-16C	0.07 ± 0.04	0.08 ± 0.04	0.06 ± 0.05	0.127	4.82^−5^ (1.58^−11^–147.13)	0.192
SCD-18C	1.23 ± 0.43	1.06 ± 0.27	1.39 ± 0.51	0.011 *	7.73 (1.32–45.25)	0.023 *

PUFAs: Polyunsaturated Fatty Acids; SFAs: Saturated Fatty Acids; MUFAs: monounsaturated fatty acids; Stearoyl-CoA desaturase 1 (SCD1) SCD-16C: C16:1(*n*-7)/C16:0; SCD-18C: C18:1(*n*-9)/C18:0; *: statistically significant difference. *p* ^1^: *p*-value of difference between means; *p* ^2^: *p*-value of logistic regression; OR: odds ratio.

**Table 3:** Adjusted logistic regression analyses of the association between high cardiovascular risk and predictor variables.

Variable	*p*-Value	High Risk OR (95%CI)
C16:0	0.030 *	1.36(1.03–1.79)
MUFA Total	0.032 *	1.52 (1.03–2.26)
C18:1n[9]	0.045 *	1.36 (1.05–1.83)
C18:3n[6]	0.051 *	0.27 (0.07–1.01)
SCD-18C	0.060	13.50 (0.90–202.51)

Logistic regression was adjusted for age, height, Homa-IR, HDL-c, and use of hypoglycemic agents. *: statistically significant difference.

**Table 4:** Correlation between Metscore, lipid, glucose, and some fatty acid profile variables.

Variable		*p*-Value	Correlation Coefficient
HOMA-IR	Metscore	0.001	0.481
HbA1c	Metscore	<0.001	0.526
VLDL-C	Metscore	<0.001	0.556
c16:0	Metscore	0.041	0.320
c18:1n[9]	Metscore	0.003	0.334
Total MUFA	Metscore	0.002	0.360
SCD-18	Metscore	0.004	0.316
HDL-C	Metscore	0.001	−0.514
QUICKI	Metscore	0.005	−0.430
Omega 6	Metscore	0.004	−0.313
c18:3n[6]	Metscore	0.003	−0.313
c18:1n[7]	QUICKI	0.013	−0.389
c18:1n[9]	QUICKI	0.002	−0.477

HOMA-IR: Homeostatic Model Assessment of Insulin Resistance; HbA1c: Hemoglobin A1c (glycated hemoglobin); VLDL-C: Very Low-Density Lipoprotein Cholesterol; Total MUFA: Total Monounsaturated Fatty Acids; SCD-18: Stearoyl-CoA Desaturase-18 (C18:1(*n*-9)/C18:0); HDL-C: High-Density Lipoprotein Cholesterol; QUICKI: Quantitative Insulin Sensitivity Check Index.

## Data Availability

The data presented in this study are available on request from the corresponding author due to ethical reasons.
